# A Rare Case of Pleural Empyema Caused by Campylobacter rectus

**DOI:** 10.7759/cureus.23205

**Published:** 2022-03-16

**Authors:** Luísa Figueiredo, José Ferrão, Catarina Ferreira, Ana Fernandes, Maria João Costa

**Affiliations:** 1 Stomatology Department, Hospital de São José - Centro Hospitalar Universitário de Lisboa Central, Lisbon, PRT; 2 Pediatric Stomatology Department, Hospital Dona Estefânia - Centro Hospitalar Universitário de Lisboa Central, Lisbon, PRT

**Keywords:** periodontal status, oral diseases, campylobacter rectus, periodontal pathogens, pleural empyema

## Abstract

*Campylobacter rectus* is considered to be a primary periodontal pathogen that is rarely identified in extraoral specimens. We report a case of pleural empyema caused by *Campylobacter rectus*: the pathogen was isolated in the drained pleural fluid sample. Since the patient had previously undergone multiple antibiotic treatments, oral cultures were highly unlikely to be positive, although poor dental hygiene appears to be the leading risk factor for *C. rectus* systemic infections. The present case illustrates that *C. rectus* can be a cause of not only periodontal disease but also pulmonary infection.

## Introduction

*Campylobacter rectus *(formerly *Wolinella recta* [1]) is a periodontal pathogen that can also be found in necrotic pulp in radicular root canals [2,3]. It requires microaerobic or anaerobic conditions for its growth, and it is rarely found in extraoral sites [4].

*Campylobacter rectus* is gram-negative, with no spores, and can be cultured in a microaerobic or anaerobic state. Its colonies are translucent, rough, flat, and nonhemolytic. The morphology of *Campylobacter rectus* is straight rod-shaped, arcuate, or “S” shape. Urease and oxidase tests are both negative [3].

*Campylobacter rectus* is part of the human oral flora and has been found in areas such as the periodontal sulcus, tongue, cheek mucosa, and saliva [5]. *Campylobacter rectus* is associated with periodontal disease [6] as its concentration is higher in diseased subgingival sites [5].

We report a case of pleural empyema caused by *Campylobacter rectus*.

This article was previously presented as a meeting poster at the 15th Biennal Congress - European Association of Oral Medicine - Virtual Congress 2021 on September 25, 2021.

## Case presentation

**Figure 1:**
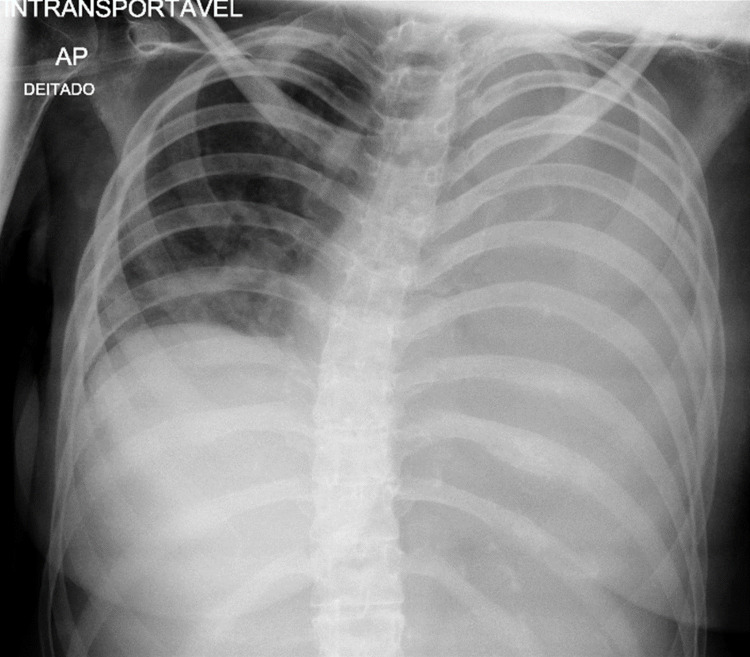
Chest X-ray showing an opacified left hemithorax.

A 37-year-old female suffering from sclerosing panencephalitis with severe cognitive and motor impairment presented in the emergency room with fever, productive cough, and prostration lasting for three days. Clinical examination performed at the time of admission showed a lethargic patient, polypneic at 26 breaths/minute, with 92% ambient air oxygen saturation. Her blood pressure was 120/102 mmHg, heart rate was 116 beats/minute, and body temperature was 37.9°C. Respiratory examination revealed mild polypneic with dullness to percussion over the left lung. Auscultation revealed decreased breath sounds in the same area, but no crackles or wheezing. The remainder of the physical examination was normal. There was no jugular venous distention. A complete blood cell count was performed, which showed anemia, neutrophilic leukocytosis, normal platelet count, increased gamma-glutamyl transferase levels, high alkaline phosphatase levels, and high C-reactive protein levels. Chest X-ray showed a nearly total opacification of the left lung field (Figure 1). The patient was then admitted for suspicion of community-acquired pneumonia.

**Figure 2:**
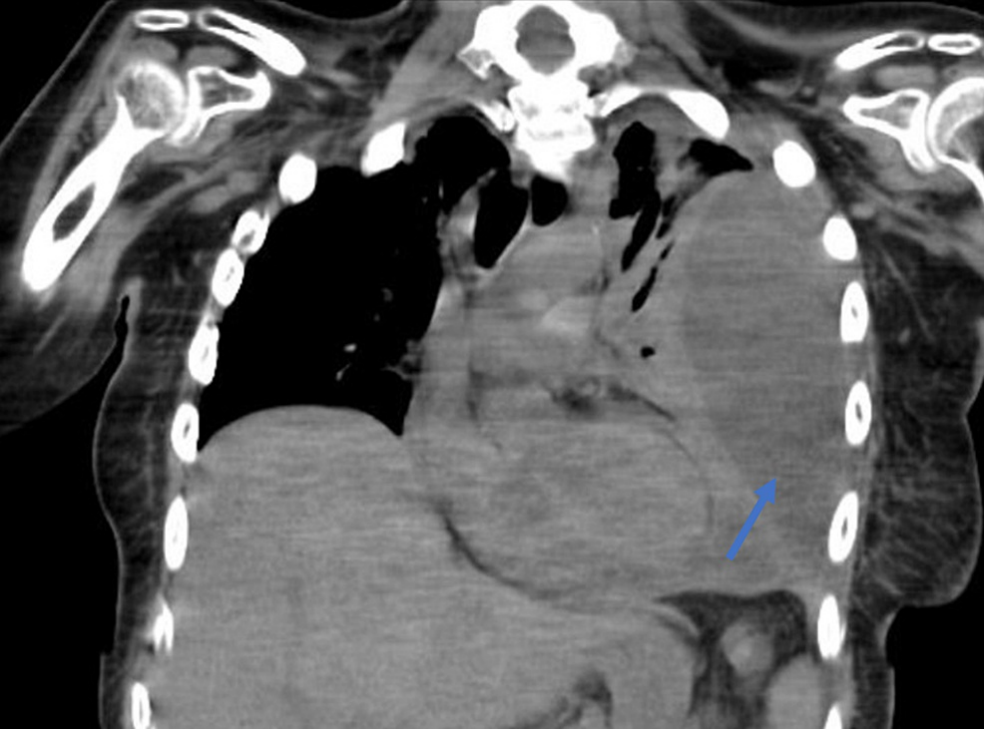
CT scan (coronal view) showing a left pulmonary empyema (blue arrow).

A computed tomography (CT) scan (without contrast) demonstrated a loculated pleural effusion, namely, in the posterolateral and anterior aspects, associated with densification of adjacent mediastinal fat, content denser than water, suggestive of empyema - pleural empyema (Figure 2).

A CT scan-guided thoracentesis was performed, and the results of the pleural fluid analysis are as follows: purulent (color); proteins, 29.6 (within the normal range); pH 7.5 (high); leucocytes, 18,124 uL (>1,000 exudate); and LDH, 169 IU/L (within the normal range). The microbiological examination of the drained fluid revealed a *Campylobacter rectus *infection.

As *C. rectus* is implicated in chronic periodontitis and has rarely been found as an extraoral specimen, it was necessary to evaluate her oral health status to identify the route of infection.

**Figure 3:**
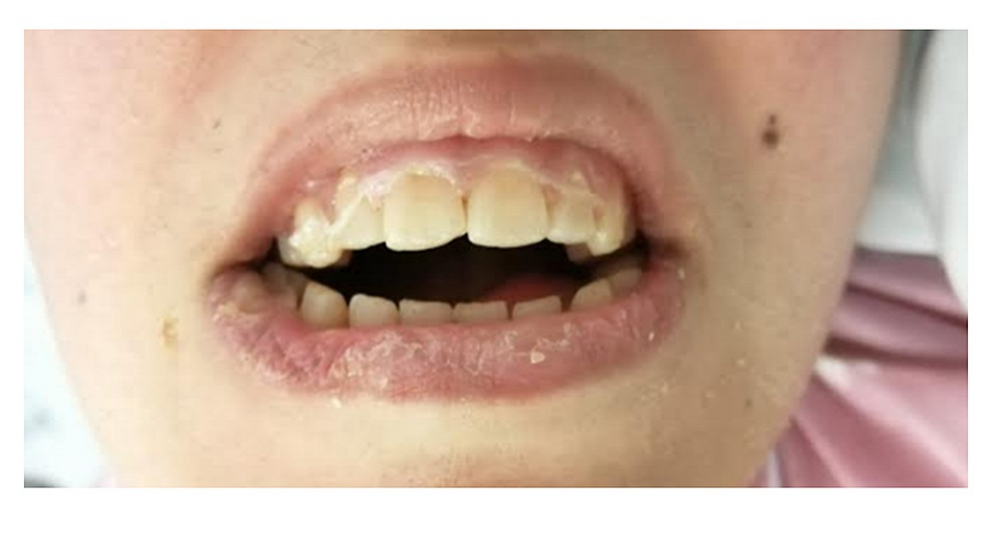
Photograph of the patient’s mouth showing the patient’s oral status. Dehydrated lips, poor oral hygiene, and dental plaque can be seen.

We were able to make an appropriate periodontal probing, finding pockets up to 6 mm, mainly in the posterior molar region. We also identified significant areas of gingival inflammation and bleeding, allowing us to make a clinical diagnosis of periodontal disease (stage III, grade A) (Figure 3).

Although based only on a limited number of studies, *C. rectus* has been shown to be sensitive to antibiotic therapy such as amoxicillin-clavulanate, cefoxitin, clindamycin, imipenem, levofloxacin, and metronidazole [1,5]. Since the patient had previously undergone over 17 days of piperacillin + tazobactam, four days of clindamycin, 21 days of meropenem, and 24 hours of amoxicillin and clavulanic acid treatment, oral cultures (collected with a Gracey curette under anaerobic conditions) were less likely to be positive, which was proven in this case.

During hospitalization time, there were complications that worsened the patient’s condition. These included bacteremia due to *Klebsiella pneumoniae *carbapenemase (KPC)-producing *Klebsiella pneumoniae*​​​​​​​, symptomatic methicillin-resistant *Staphylococcus aureus* (MRSA) infection of the percutaneous endoscopic gastrostomy (PEG) site, *Enterococcus faecium-*related cystitis, and SARS-CoV-2 infection.

The medical team was able to stabilize her condition after 117 days of hospitalization, and she was included in a home-based palliative care program.

## Discussion

*Campylobacter rectus* is a difficult organism to culture and identify:* *it requires an anaerobic atmosphere for optimal isolation, although the necessary gas composition for its growth is variable according to the literature [5].

Contrary to published literature [5], this patient did not present any oral abscess. As a result, sample collection and storage were a challenge. We used a Gracey curette and an anaerobic transport solution (a mineral salt-based semisolid anaerobic medium with added cysteine) to provide an oxygen-reduced environment. The tube has a 25-mm-wide opening, which makes it easier to place large instruments into it.

In this very rare case, a focal pulmonary infection of periodontal origin is the most probable clinical diagnosis. We speculate that the orally hosted organism disseminated to the pleural space by aspiration since the patient had a prior history of aspiration pneumonia.

## Conclusions

*Campylobacter rectus* is an infrequent anaerobic pathogen of known periodontal origin. This case report shows an extraoral site of *C. rectus*, presenting as a pleural empyema. Culture samples confirmed a *Campylobacter rectus* infection.

There are very few cases reporting extraoral infections caused by *C. rectus*. Although we were not able to prove it, we believe that dissemination from the oral cavity to the pleural space via/through aspiration is the most probable cause.

The oral cavity can be the origin of potentially fatal systemic infections, and all clinics should be aware of that. The oral cavity should not be neglected when in search of an infection origin in order to treat accordingly.

## References

[1] Rams TE, Feik D, Slots J (1993). Campylobacter rectus in human periodontitis. Oral Microbiol Immunol.

[2] Ogata T, Urata T, Nemoto D, Hitomi S (2017). Thoracic empyema caused by Campylobacter rectus. J Infect Chemother.

[3] Zhu X, Yu S, Kang Q, Qiu Y, Tian M, Cao E (2021). Campylobacter rectus infection leads to lung abscess: a case report and literature review. Infect Drug Resist.

[4] Noël A, Verroken A, Belkhir L, Rodriguez-Villalobos H (2018). Fatal thoracic empyema involving Campylobacter rectus: a case report. Anaerobe.

[5] Mahlen SD, Clarridge JE 3rd (2009). Oral abscess caused by Campylobacter rectus: case report and literature review. J Clin Microbiol.

[6] Matsumoto R, Himeji D, Shiiba R (2021). Thoracic empyema caused by Campylobacter rectus: a case report. Intern Med.

